# WeChat-based education and rehabilitation program in unprotected left main coronary artery disease patients after coronary artery bypass grafting: an effective approach in reducing anxiety, depression, loss to follow-up, and improving quality of life

**DOI:** 10.1590/1414-431X202010370

**Published:** 2021-02-12

**Authors:** Chongyi Ma, Bo Wang, Xiaomeng Zhao, Fan Fu, Lei Zheng, Guorong Li, Qingfeng Guo

**Affiliations:** 1 Department of Cardiovascular Surgery, Second Affiliated Hospital of Harbin Medical University, Harbin, Heilongjiang, China; 2 Ministry of Nursing, 4th Affiliated Hospital of Harbin Medical University, Harbin, Heilongjiang, China

**Keywords:** WeChat-based education and rehabilitation program, ULMCAD, Anxiety and depression, Quality of life, Loss to follow-up

## Abstract

This study aimed to investigate the effect of WeChat-based education and rehabilitation program (WERP) on anxiety, depression, health-related quality of life (HRQoL), major adverse cardiac/cerebrovascular events (MACCE)-free survival, and loss to follow-up rate in unprotected left main coronary artery disease (ULMCAD) patients after coronary artery bypass grafting (CABG). In this randomized controlled study, 140 ULMCAD patients who underwent CABG were randomly assigned to WERP group (n=70) or control care (CC) group (n=70). During the 12-month intervention period, anxiety and depression (using hospital anxiety and depression scale (HADS)) and HRQoL (using 12-Item Short-Form Health Survey (SF-12)) were assessed longitudinally. During the total 36-month follow-up period (12-month intervention and 24-month non-intervention periods), MACCE and loss to follow-up were recorded. During the intervention period, HADS-anxiety score at month 9 (M9) (P=0.047) and month 12 (M12) (P=0.034), anxiety rate at M12 (P=0.028), and HADS-D score at M12 (P=0.048) were all reduced in WERP group compared with CC group. As for HRQoL, SF-12 physical component summary score at M9 (P=0.020) and M12 (P=0.010) and SF-12 mental component summary score at M9 (P=0.040) and M12 (P=0.028) were all increased in WERP group compared with CC group. During the total follow-up period, WERP group displayed a trend of longer MACCE-free survival than that in CC group but without statistical significance (P=0.195). Additionally, loss to follow-up rate was attenuated in WERP group compared with CC group (P=0.033). WERP serves as an effective approach in optimizing mental health care and promoting life quality in ULMCAD patients after CABG.

## Introduction

Unprotected left main coronary artery disease (ULMCAD) represents a highly lethal condition with more than 50% stenosis of the left main coronary artery without patent bypass graft to the left ventricular myocardium (1,2). For several decades, coronary artery bypass grafting (CABG) has been recommended as the standard revascularization procedure in the treatment of ULMCAD patients based on early clinical trials demonstrating a conferred survival benefit (3). However, owing to daily fatigue and cardiac symptoms (such as pain and dyspnea), ULMCAD patients frequently experience psychological distress including anxiety and depression after CABG (4,5). Anxiety and depression are related to lower compliance with treatment/lifestyle changes, increased risk of major adverse cardiac events, elevated rates of all-cause mortality, and diminished quality of life in ULMCAD patients after CABG (6,7). Therefore, effective psychological and psychopharmacological interventions for attenuating anxiety and depression, promoting functional recovery, as well as improving health-related quality of life (HRQoL) are essential in ULMCAD patients after CABG.

Non-pharmacological interventions such as exercise therapy, target therapy of psychosocial needs, or education are known to provide emotional support, reduce anxious/depressive symptoms, and improve HRQoL in coronary artery diseases patients after CABG (8-10). As for ULMCAD patients after CABG, only one previous study showed that a comprehensive rehabilitation and intensive education program decreases anxiety and depression and improves HRQoL (11). However, most of the reported non-pharmacological interventions are center-based (that is limited by financial, transport, or disability constrains) or telephone-based (that is limited by oral information and high rates of missing contact) (12,13). Therefore, a rapid-delivery easy-to-use tool with flexibility and timely feedback is vital for assistance with non-pharmacological interventions.

WeChat (https://weixin.qq.com/, Tencent Corporation, China), a multi-functional social media platform, is emerging as an essential and daily communication tool for Chinese people with over 1.13 billion monthly active users from a wide range of age groups in 2019 (14). Owing to its large user population, powerful multiple functions, and integration into everyday life, WeChat-based non-pharmacological interventions have the potential of being a valuable strategy for mental health care and secondary prevention in ULMCAD patients after CABG. However, no relevant research has been reported on this topic. Therefore, the present randomized controlled study established a WeChat-based education and rehabilitation program (WERP) consisting of health education, rehabilitation guidance, exercise supervision, and psychological care, aiming to investigate its effect on anxiety, depression, HRQoL, major adverse cardiac/cerebrovascular events (MACCE)-free survival, and loss to follow-up rate in ULMCAD patients after CABG.

## Material and Methods

### Participants

From July 2015 to June 2016, 140 ULMCAD patients who underwent CABG in our hospital were consecutively recruited in this randomized, enrolled study. The inclusion criteria were as follows: 1) angiographically confirmed ULMCAD, which was defined as the left main artery luminal narrowing of more than 50% without patent bypass grafts to its branches (2); 2) age ≥18 years; 3) about to receive CABG; 4) voluntary to comply with study follow-up protocol and able to be regularly followed up; and 5) proficient in using WeChat mobile application (app), which was defined as the patient being able to independently use the basic functions in WeChat app, such as receiving and sending information, making video calls, and reading articles delivered. The exclusion criteria included: 1) had contraindications to undergo CABG; 2) presented with cardiogenic shock; 3) complicated with poorly controlled comorbidities; 4) concomitant with malignant tumors; 5) known severe mental illness (e.g., schizophrenia); and 6) pregnant or breast-feeding women. This study was approved by the Ethics Committee of our hospital, and all participants signed the informed consents.

### Randomized grouping

After enrollment, eligible patients were randomly allocated to two groups: WERP group (n=70) or control care group (CC group, n=70), using the block randomization method with a block size of 4 and the ratio of 1:1. Randomization allocation table was created by an independent analyst using SAS 9.0 (SAS Institute, Inc., USA). Sequential numbers were assigned to 140 sealed, opaque envelopes. These envelopes contained the assigned intervention group for each patient, based on the randomization allocation table. Envelopes were opened by the investigator right after CABG in sequential order for each qualifying patient. During hospitalization, surgeons did not know which group the patients were included in, while the nursing team involved in this study knew which group the patients were included in.

### Baseline data collection

Baseline clinical data of enrolled patients were documented in detail, mainly including the following: 1) demographics: age, gender, body mass index (BMI), education duration, marital status, employment status before surgery, current smoker; 2) comorbidities: hypertension, hyperlipidemia, diabetes, chronic lung disease, chronic renal failure; 3) medical histories: family history of CAD, previous myocardial infarction, previous stroke, previous heart failure, previous percutaneous coronary intervention (PCI); 4) clinical manifestation: stable angina, unstable angina, left ventricular ejection fraction (LVEF) level, disease extent as well as involvement extent; 5) CABG type: on-pump and off-pump; 6) medications use after CABG: beta blockers, angiotensin converting enzyme inhibitors/ angiotensin receptor blocker (ACEI/ARB), calcium channel blocker (CCB), statins, fibrates, niacins and nitrates; and 7) use of psychotropic medications. All enrolled patients received on-pump or off-pump CABG, and graft selection and the choice of on- or off-pump surgery was made by the attending surgeon. During hospitalization, no deteriorated events occurred in ULMCAD patients who underwent CABG. Notably, the time interval between enrollment and randomization was short, therefore, the baseline characteristics of patients were not repeatedly collected.

### Interventions

All recruited ULMCAD patients received CABG. After CABG, all patients received post-CABG care. CABG, post-CABG care, and secondary prevention were conducted as usual practice and were not intervened. As for psychotropic medications, patients with severe or moderate anxiety and depression received psychotropic medications according to their clinical status. The study intervention with WERP program or CC program was initiated after patients were discharged from the hospital and lasted for 12 months.

#### WERP group

The WERP program was conducted by a team of nurses who received special training about WERP. WERP was carried out on the WeChat app (Tencent Corporation, China), which supported the rapid delivery of free (requiring a small amount of network traffic) voice message, video, picture, and text over the network. After enrollment, patients were invited to attend a session during which these nurses introduced WERP in detail. Then a WeChat group chat was organized by the nurses, and patients who were recruited in the study at the same month were invited into the same WeChat group chat. WERP was performed for a total of 12 months, which included four parts: 1) Health education: the nurses delivered the disease-related health educational courses into the WeChat group chat. In each course, the nurses explained disease-related knowledge including post-CABG medicine management, risk factor management, smoking cessation management, nutrition intake management, physical activity, and secondary prevention management. All courses were delivered in the form of short videos and updated weekly for a total of 12 months. After each delivery of video courses in the WeChat group chat, patients were required to learn the contents of videos carefully and reply “received” when finishing the course. If there was any question about the video courses, the patient was encouraged to contact the nurses in the WeChat group chat or in one-to-one chatting model if necessary, and then the nurses were responsible for replying to patients’ questions promptly In addition, if patients needed a clinical appointment, they could inform the nurses in the WeChat group chat who would schedule a clinic appointment for them. 2) Rehabilitation guidance: the rehabilitation guidance was focused on exercise, including aerobic exercise, resistance training, flexibility training, and balance training. The rehabilitation course was also weekly delivered in the form of short video courses for 12 months. All courses were formed referring to expert consensus (15). In each video course, the nurses demonstrated the exercise posture in detail and emphasized the key points and attention points. Also, if patients had any question related to the video courses, counseling in the WeChat group chat or in one-to-one chatting model was encouraged. Furthermore, patients were asked to contact the nurses if they experienced any symptoms during or after exercising. 3) Exercise supervision: before discharge from the hospital, patient clinical conditions were comprehensively evaluated, and the exercise cardiopulmonary tests were carried out to determine the safe range of exercise training, conduct risk stratification, and develop individualized exercise prescriptions for patients. After discharge, patients were required to execute the individualized exercise prescription and weekly report their progress in the WeChat group chat for 12 months. Family members of patients were advised to monitor the patient's exercise progress by video recording and to post the video on the WeChat group chat, and the nurses were responsible for supervising the progress and urging patients promptly. 4) Psychological care: an individualized nurse-led counseling via the video call on the WeChat was conducted every two weeks by the nurses for 12 months. In each counseling, the nurses would intimately chat with patients to know about their life situation, health conditions, and mental status. Patients were encouraged to pour out recent problems and troubles, and the nurses tried to comfort patients, understand the patients’ troubles, and give them support. If patients presented with negative emotions, the nurses would help them release negative emotions and encourage patients to cooperate with study intervention.

#### CC group

Patients in CC group were given disease-related health education (only once) before discharge from the hospital, including post-CABG medicine management, risk factor management, smoking cessation management, nutrition intake management, physical activity, and secondary prevention management. Also, an individualized exercise prescription was developed for every patient. After patients were discharged from the hospital, the trained nurse would make a phone call to patients every month for a total of 12 months to provide rehabilitation guidance and medical psychological counseling (each lasted about 30 min), which was given to the patients based on individual needs.

#### Anxiety, depression, and HRQoL assessment

Study assessments were conducted at baseline (M0), month 3 (M3), M6, M9, and M12, including anxiety, depression, and HRQoL assessments. Anxiety and depression were evaluated by Hospital Anxiety and Depression Scale (HADS). The HADS consisted of two subscales: HADS-anxiety (HADS-A) scale and HADS-depression (HADS-D) scale, and each subscale included 7 questions, which were scored as 0-3 points individually, resulting in a total score of 21. Anxiety/depression was defined as HADS-A/HADS-D score >7 (16). HRQoL was assessed by 12-Item Short-Form Health Survey (SF-12), which was previously validated for the assessment of HRQoL in Chinese (17). There are two subscales in the SF-12: a) Physical Component Summary (PCS), an index of overall physical functioning; and b) Mental Component Summary (MCS), an index of emotional and mental health. Higher SF-12 PCS or SF-12 MCS scores indicate better self-perceived health. The evaluation was managed at the research institute as prescheduled. Specific training on the measurement was conducted for investigators responsible for data collection.

### Follow-up and MACCE assessment

Follow-up for all patients was managed according to the 2011 ACCF/AHA Guideline for Coronary Artery Bypass Graft Surgery (18). All patients were constantly followed up by telephone contact or clinical visits until the occurrence of MACCE, or the completion of the total 36-month follow-up. MACCE was defined as a composite of death, myocardial infarction, stroke, or repeat revascularization (19). Patients lost to follow-up were censored on the date of last visit in the MACCE analysis, and the last observation carried forward (LOCF) method was used to process the missing measurement data.

### Sample size calculation

The required minimum sample size was estimated based on a prediction of anxiety rate of 12% at M12 in the WERP group and 35% at M12 in the CC group. Using PASS V11.0 software (NCSS, USA), with a power of 80%, a two-sided 5% level of significance (α), and Fisher's exact test, the required minimum sample size was 60 participants in each group. Assuming an approximately 15% attrition rate, the final sample size was inflated to 70 patients in each group.

### Statistical analysis

All patients were included in the final analysis based on the intention-to-treat (ITT) principle. All statistical analyses and figure plotting were performed using SPSS 24.0 statistical software (IBM, USA) and GraphPad Prism 7.02 software (GraphPad Software Inc., USA). Data are reported as means±SD, or count (percentage). Comparisons between two groups were determined by the Student's *t-*test or chi-squared test. MACCE-free survival was calculated from the date of CABG to the date of MACCE occurrence, which is reported using Kaplan-Meier curve, and the difference of MACCE-free survival between the two groups was determined by the log-rank test. All tests were 2-tailed and P<0.05 indicated a significant difference.

## Results

### Study flow

**Figure 1:**
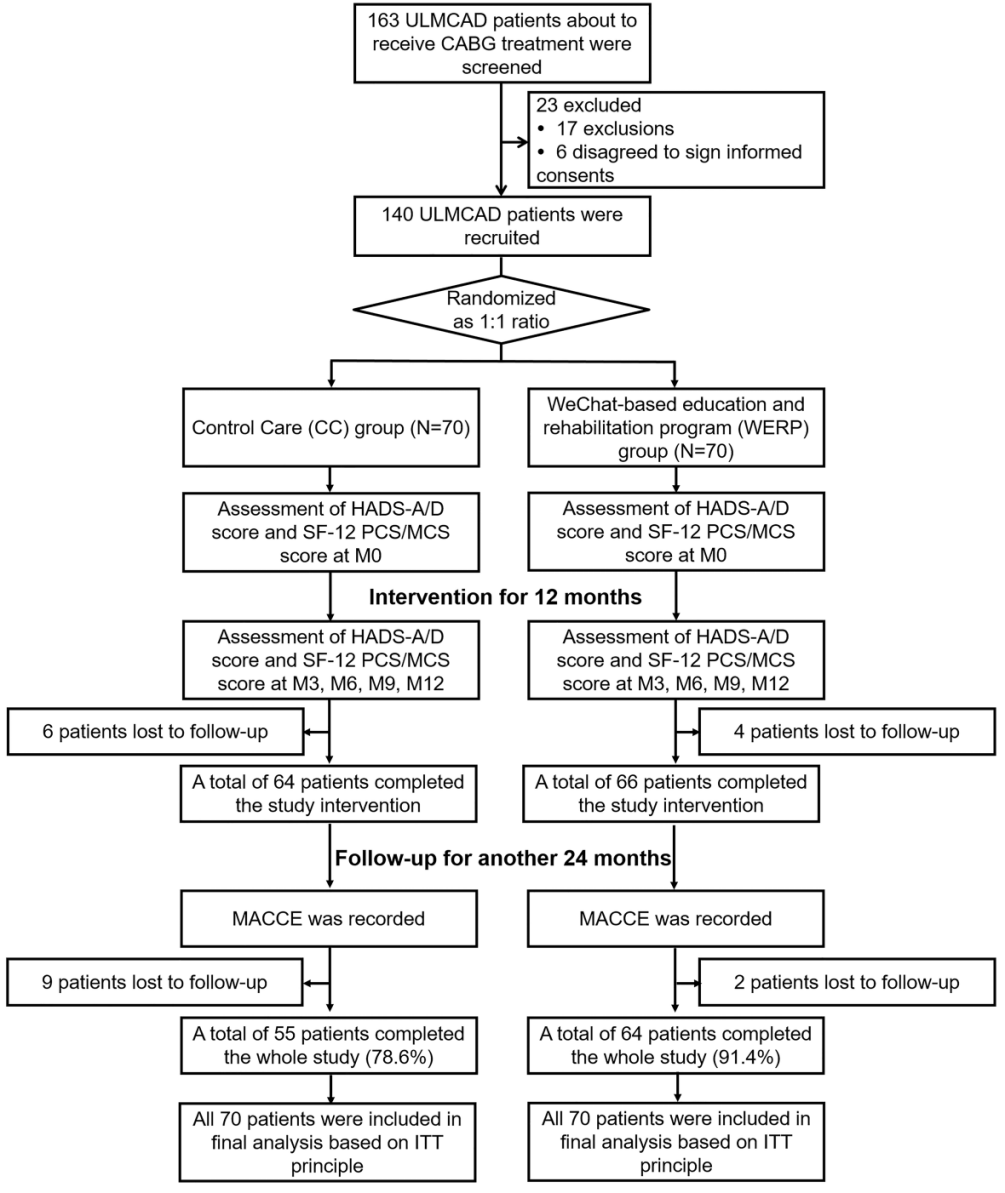
Flow chart of study. ULMCAD: unprotected left main coronary artery disease; CABG: coronary artery bypass graft; HADS-A/D: hospital anxiety and depression scale-anxiety/depression; SF-12 PCS/MCS: 12-item short-form health survey physical component summary/mental component summary; M0: baseline; M3, month 3; M6: month 6; M9: month 9; M12: month 12; MACCE: major adverse cardiac and cerebrovascular events; ITT: intention-to-treat.

Initially, 163 ULMCAD patients who were about to receive CABG treatment were screened, among which 23 patients were excluded (17 patients did not meet inclusion criteria or met exclusion criteria, 6 patients disagreed to sign informed consents) (Figure 1). The remaining 140 ULMCAD patients were randomly assigned to CC group (n=70) or WERP group (n=70) in a 1:1 ratio. Then, HADS-A/D score and SF-12 PCS/MCS score at M0 were assessed in both CC and WERP groups. During the 12-month intervention period, HADS-A/D score and SF-12 PCS/MCS score were assessed at M3, M6, M9, and M12 in both CC and WERP groups. Meanwhile, 6 patients in CC group and 4 patients in WERP group were lost to follow-up within the intervention period, which resulted in 64 and 66 patients who completed the study intervention in CC group and WERP group, respectively. For the subsequent 24 months (non-intervention period), no intervention was conducted in CC and WERP groups while follow-up was continued. During the non-intervention period, 9 patients in CC group and 2 in WERP group were lost to follow-up. Finally, a total of 55 (78.6%) patients in CC group and 64 (91.4%) in WERP group completed the whole study. In the final analysis, all 70 patients in CC group and 70 patients in WERP group were included based on the ITT principle. During the total follow-up period (intervention and non-intervention periods), MACCE were recorded.

### Comparison of baseline characteristics

The mean age was 65.1±7.5 years in CC group and 65.2±7.3 years in WERP group, and there were 24.3% females/75.7% males in CC group and 17.1% females/82.9% males in WERP group (Table 1). Regarding demographics, comorbidities, medical histories, and clinical manifestations, no differences were found between WERP group and CC group.

**Table 1:** Baseline characteristic of unprotected left main coronary artery disease patients after coronary artery bypass graft that received the WeChat-based education and rehabilitation program (WERP) and the control care (CC).

Items	CC group (n=70)	WERP group (n=70)	P value
Age (years)	65.1±7.5	65.2±7.3	0.946
Gender			0.297
Female	17 (24.3)	12 (17.1)	
Male	53 (75.7)	58 (82.9)	
BMI (kg/m^2^)	24.1±2.6	24.4±3.2	0.511
Education duration (years)	8.4±4.7	8.8±3.6	0.628
Marital status			0.735
Single/divorced/widowed	36 (51.4)	38 (54.3)	
Married	34 (48.6)	32 (45.7)	
Employed before surgery			0.769
No	61 (87.1)	62 (88.6)	
Yes	9 (12.9)	8 (11.4)	
Current smoker	18 (25.7)	21 (30.0)	0.572
Hypertension	47 (67.1)	45 (64.3)	0.722
Hyperlipidemia	28 (40.0)	23 (32.9)	0.380
Diabetes	14 (20.0)	18 (25.7)	0.421
Chronic lung disease	3 (4.3)	4 (5.7)	0.698
Chronic renal failure	2 (2.9)	2 (2.9)	1.000
Family history of CAD	12 (17.1)	10 (14.3)	0.642
Previous myocardial infarction	14 (20.0)	16 (22.9)	0.680
Previous stroke	3 (4.3)	5 (7.1)	0.466
Previous heart failure	3 (4.3)	4 (5.7)	0.698
Previous PCI	10 (14.3)	7 (10.0)	0.438
Clinical presentation			0.398
Unstable angina	38 (54.3)	33 (47.1)	
Stable angina	32 (45.7)	37 (52.9)	
LVEF level			0.410
≥50%	57 (81.4)	53 (75.7)	
<50%	13 (18.6)	17 (24.3)	
Disease extent			0.190
Left main only	3 (4.3)	1 (1.4)	
Left main + 1 vessel disease	1 (1.4)	6 (8.6)	
Left main + 2 vessel disease	14 (20.0)	15 (21.4)	
Left main + 3 vessel disease	52 (74.3)	48 (68.6)	
Distal bifurcation involvement	48 (68.6)	49 (70.0)	0.855
Right CAD involvement	49 (70.0)	57 (81.4)	0.115
CABG type			0.730
On-pump	66 (94.3)	65 (92.8)	
Off-pump	4 (5.7)	5 (7.2)	
Medication use after CABG			
Aspirin	53 (75.7)	50 (71.4)	0.565
Beta blockers	37 (52.8)	39 (55.7)	0.735
ACEI/ARB	30 (42.8)	33 (47.1)	0.610
CCB	20 (28.5)	21 (30.0)	0.852
Statins	21 (30.0)	19 (27.1)	0.708
Fibrates	15 (21.4)	19 (27.1)	0.430
Niacins	13 (18.6)	9 (12.8)	0.353
Nitrates	10 (14.3)	8 (11.4)	0.614
HADS-A score	7.0±3.6	7.1±2.9	0.817
Anxiety	23 (32.9)	26 (37.1)	0.595
HADS-D score	6.6±3.4	6.9±3.4	0.585
Depression	20 (28.6)	22 (31.4)	0.712
SF-12 PCS score	35.7±8.5	36.0±7.0	0.820
SF-12 MCS score	42.9±8.5	42.4±11.3	0.775
Use of psychotropic medications	11 (15.7)	8 (11.4)	0.459

Data are reported as number with percent in parentheses or mean±SD. Student's *t-*test or chi-squared test was used for statistical analysis. BMI: body mass index; CAD: coronary artery disease; PCI: percutaneous coronary intervention; LVEF: left ventricular ejection fraction; CABG: coronary artery bypass graft; ACEI/ARB: angiotensin converting enzyme inhibitors/ angiotensin receptor blocker; CCB: calcium channel blocker; HADS-A: hospital anxiety and depression scale-anxiety; HADS-D: hospital anxiety and depression scale-depression; SF-12 PCS: 12-Item Short-Form Health Survey Physical Component Summary; SF-12 MCS: 12-Item Short-Form Health Survey Mental Component Summary.

### Comparison of HADS-A score and anxiety rate at different time points

**Figure 2:**
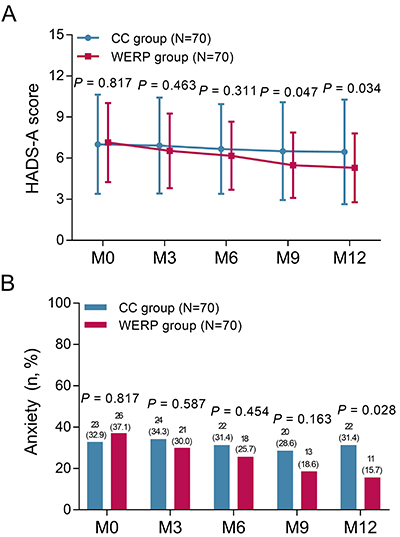
Comparison of HADS-A score (**A**) and anxiety rate (**B**) at month (M)0, M3, M6, M9, and M12 between WERP group and CC group of unprotected left main coronary artery disease patients after coronary artery bypass graft. HADS-A: hospital anxiety and depression scale-anxiety; WERP: WeChat-based education and rehabilitation program; CC: control care. Data are reported as means±SD or count and percentage (Student's *t-*test).

HADS-A score at M0 (P=0.817), M3 (P=0.463), and M6 (P=0.311) were similar between WERP group and CC group, while HADS-A score at M9 (5.5±2.4 *vs* 6.5±3.6, P=0.047) and M12 (5.3±2.5 *vs* 6.5±3.8, P=0.034) were lower in WERP group than in CC group (Figure 2A). No difference in anxiety rate at M0 (P=0.817), M3 (P=0.587), M6 (P=0.454), or M9 (P=0.163) was found between WERP group and CC group, while anxiety rate at M12 (15.7 *vs* 31.4%, P=0.028) was attenuated in WERP group compared with that in CC group (Figure 2B).

### Comparison of HADS-D scores and depression rates at different time points

**Figure 3:**
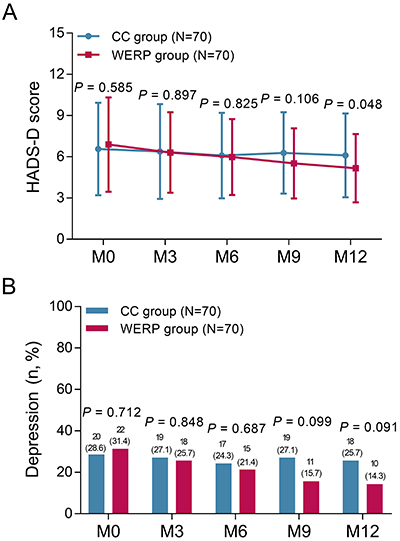
Comparison of HADS-D score (**A**) and depression rate (**B**) at month (M)0, M3, M6, M9, and M12 between WERP group and CC group of unprotected left main coronary artery disease patients after coronary artery bypass graft. HADS-D: hospital anxiety and depression scale-depression; WERP: WeChat-based education and rehabilitation program; CC: control care. Data are reported as means±SD or count and percentage (Student's *t-*test).

HADS-D scores at M0 (P=0.585), M3 (P=0.897), M6 (P=0.825), and M9 (P=0.106) were similar between WERP group and CC group, while HADS-D score at M12 (5.2±2.5 *vs* 6.1±3.1, P=0.048) was lower in WERP group than that in CC group (Figure 3A). Meanwhile, depression rates at M0 (P=0.712), M3 (P=0.848), M6 (P=0.687), M9 (P=0.099), and M12 (P=0.091) were not different between the two groups (Figure 3B).

### Comparison of SF-12 scores at different time points

**Figure 4:**
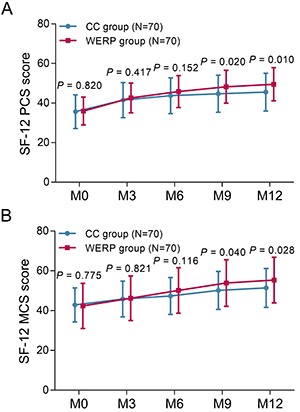
Comparison of SF-12 PCS score (**A**) and SF-12 MCS score (**B**) at month (M)0, M3, M6, M9, and M12 between WERP group and CC group of unprotected left main coronary artery disease patients after coronary artery bypass graft. Data are reported as means±SD (Student's *t-*test). SF-12 PCS and MCS: 12-Item Short-Form Health Survey Physical Component Summary and Mental Component Summary; WERP: WeChat-based education and rehabilitation program; CC: control care.

SF-12 PCS scores at M0 (P=0.820), M3 (P=0.417), and M6 (P=0.152) were similar between WERP group and CC group, while SF-12 PCS scores at M9 (48.3±8.3 *vs* 44.7±9.4, P=0.020) and M12 (49.5±8.4 *vs* 45.5±9.6, P=0.010) were higher in WERP group than that in CC group (Figure 4A). Furthermore, SF-12 MCS scores at M0 (P=0.775), M3 (P=0.821), and M6 (P=0.116) were not different between the two groups, while SF-12 MCS scores at M9 (53.9±11.7 *vs* 50.2±9.5, P=0.040) and M12 (55.4±11.5 *vs* 53.9±11.7, P=0.028) were elevated in WERP group compared with CC group (Figure 4B).

### Comparison of MACCE-free survival

**Figure 5:**
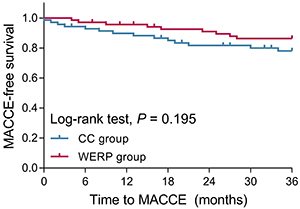
Kaplan-Meier curve showing major adverse cardiac and cerebrovascular events (MACCE)-free survival within total follow-up period of WERP group and CC group of unprotected left main coronary artery disease patients after coronary artery bypass graft. WERP: WeChat-based education and rehabilitation program; CC: control care.

As MACCE was one of the main concerns regarding clinical outcome in ULMCAD patients after CABG, we compared the MACCE-free survival between WERP group and CC group, and we observed that there was no statistical difference of MACCE-free survival between groups (Figure 5). However, the WERP group showed a trend of longer MACEE-free survival than CC group (P=0.195) (Figure 5). Furthermore, we also compared MACCE occurrence (death, myocardial infarction, repeat revascularization, or stroke) between groups and no difference was found (P=0.254, death (P=0.613), myocardial infarction (P=0.753), repeat revascularization (P=0.648), stroke (P=1.000) (Table 2).

**Table 2:** Major adverse cardiac/cerebrovascular events (MACCE) of unprotected left main coronary artery disease patients after coronary artery bypass graft that received the WeChat-based education and rehabilitation program (WERP) and the control care (CC) within 3 years.

Items	CC group (n=70)	WERP group (n=70)	P value
MACCE	14 (20.0)	9 (12.9)	0.254
Death	10 (14.3)	8 (11.4)	0.613
Myocardial infarction	5 (7.1)	6 (8.6)	0.753
Repeat revascularization	3 (4.3)	2 (2.8)	0.648
Stroke	3 (4.3)	3 (4.3)	1.000

Data are reported as number and percent (Student’s *t*-test).

### Comparison of loss to follow-up rate

**Figure 6:**
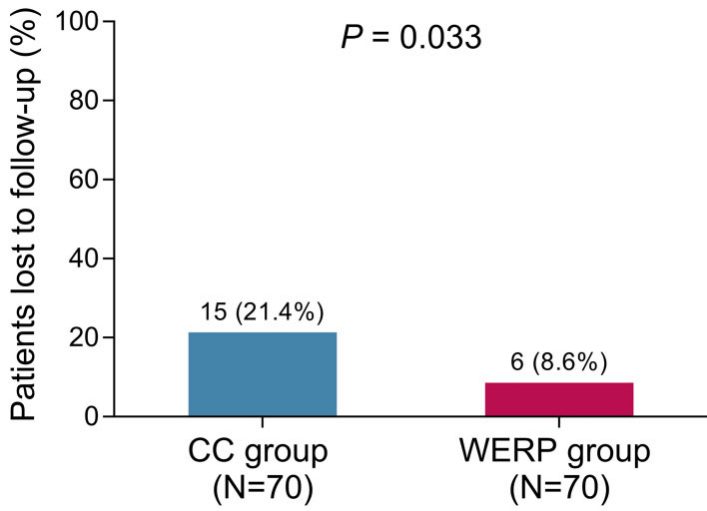
Loss of follow-up rate within total follow-up period of WERP group and CC group of unprotected left main coronary artery disease patients after coronary artery bypass graft. WERP: WeChat-based education and rehabilitation program; CC: control care. Data are reported as count and percentage (Student's *t-*test).

In WERP group, 4 patients during the intervention period and 2 patients during the non-intervention period were lost to follow-up. In CC group, 6 patients during intervention period and 9 patients during non-intervention period were lost to follow-up. By comparison, the loss to follow-up rate was lower in WERP group than that in CC group (8.6 *vs* 21.4%, P=0.033) (Figure 6).

## Discussion

Anxiety and depression are common and persistent psychological disorders in CAD patients, which in turn are linked to increased risk of worse cardiac outcomes and declined HRQoL (7). Accumulated evidence in the literature highlights that rehabilitation programs traditionally delivered in center-based or telephone-based settings are effective in improving health behaviors and mental health in CAD patients after CABG (8-10). For instance, one hospital-based physical and psycho-educational rehabilitation program decreased depression and exhibited positive effects on physical activity in CAD patients after CABG (9). Another study reports that intensive telephone-based care program relieves depression and contributes to favorable overall survival in CAD patients (10). While for ULMCAD patients who underwent CABG, only one study showed that the hospital-based comprehensive rehabilitation and intensive education program reduced anxiety and depression, and increased HRQoL in these patients (11). However, center-based or telephone-based rehabilitation programs are limited by time, location, financial burden, and inconvenience (12,13). Therefore, more effective and convenient methods for delivering rehabilitation programs are needed in ULMCAD patients who underwent CABG.

WeChat-based interventions as innovative methods for delivering rehabilitation programs have been increasingly attracting attention (20). In the present study, we designed the WeChat-based rehabilitation program WERP, which involves health education, rehabilitation guidance, exercise supervision, and psychological care. We found that WERP decreased HADS-A score at M9 and anxiety rate at M12, meanwhile WERP reduced HADS-D score at M12. These indicated that WERP was effective in relieving anxiety and depression in ULMCAD patients after CABG. Possibly, with advantages such as convenience, flexibility, and easiness the WERP program helped patients with the understanding of the disease-related knowledge and rehabilitation exercise in detail, as well as monitored whether patients complied with their medicine prescriptions and performed exercise according to procedures, thereby, strengthening self-care abilities and improving physical recovery (13). Furthermore, through the WeChat group chat or one-to-one chatting model, if patients had any discomfort or questions, they could receive counseling and solutions at a convenient time from nurses/professionals to actively adjust their functional exercises, lifestyle habits, diet, and mood. In addition, the communication between peers regarding secondary prevention experience provided emotional support, encouragement, and comfort to each other, alleviating negative emotions (13). On the other hand, CC was delivered via telephone call, which might not be timely and effective due to confusing descriptions about complications by patients, missing calls, etc. (13). Taken together the findings indicated that WERP was effective in attenuating anxiety and depression in ULMCAD patients after CABG.

As for assessment of self-reported HRQoL, SF-12 is one of the most commonly applied instruments (21). In the present study, we found that WERP increased both SF-12 PCS and MCS scores in ULMCAD patients after CABG, indicating that WERP improved HRQoL. As mentioned above, the WeChat protocol provided timely solutions regarding patients’ questions and offered necessary emotional support to patients, which enhanced self-management of disease, built-up confidence, and improved physical function and HRQoL (13).

In addition, we found that WERP showed a trend for prolonging MACCE-free survival in ULMCAD patients after CABG but without statistical significance. The possible explanations for the lack of significance might be the short follow-up duration and few ULMCAD patients with MACCE, which might markedly decrease statistical power. Besides, WERP attenuated loss to follow-up rate in ULMCAD patients after CABG. Perhaps the advantages explained above could cause WERP to increase the adherence to rehabilitation programs and reduce loss to follow-up rate compared with CC. Of note, the different frequency and availability of rehabilitation guidance and medical psychological counseling by nurses between CC (monthly) and WERP (weekly) might impact our results. It could be speculated that it actually took nurses more time and resources for performing CC compared WERP due to frequently missing calls, difficulties in collecting information about rehabilitation guidance via phone call, and imprecise descriptions about complications by patients. However, the evaluation of total time and resources spent in WERP and CC needs further investigation.

In spite of the interesting findings of the present study, several shortcomings should be noted. First, ULMCAD patients who were lost to follow-up were censored on the date of last visit for the MACCE analysis, which might cause potential bias. Second, anxiety and depression were only assessed by HADS scale, and HRQoL was only evaluated by SF-12, which needed other anxiety, depression and HRQoL assessment tools for further validation. Third, since the study was not a blinded trial, both the patients and the nurses were aware of intervention assignment, which was subject to biases in the nurses’ assessment and patents’ self-reported outcomes. Fourth, the effect of WERP on patient adherence to pharmacological and/or non-pharmacological measures and to secondary prevention drugs was not evaluated; thus, further study is needed. Lastly, enrolled patients were required to be proficient in using WeChat mobile application, which might result in selection bias.

In conclusion, WERP might be an effective approach to relieve anxiety and depression, improve HRQoL, as well as decrease loss to follow-up rate in ULMCAD patients who underwent CABG, which offered new insight for optimizing mental health care and promoting life quality of these patients.

## References

[1] Collet C, Capodanno D, Onuma Y, Banning A, Stone GW, Taggart DP (2018). Left main coronary artery disease: pathophysiology, diagnosis, and treatment. Nat Rev Cardiol.

[2] Deb S, Wijeysundera HC, Ko DT, Tsubota H, Hill S, Fremes SE (2013). Coronary artery bypass graft surgery vs percutaneous interventions in coronary revascularization: a systematic review. JAMA.

[3] Yusuf S, Zucker D, Peduzzi P, Fisher LD, Takaro T, Kennedy JW (1994). Effect of coronary artery bypass graft surgery on survival: overview of 10-year results from randomised trials by the coronary artery bypass graft surgery trialists collaboration. Lancet.

[4] Sardinha A, Araujo CG, Soares-Filho GL, Nardi AE (2011). Anxiety, panic disorder and coronary artery disease: issues concerning physical exercise and cognitive behavioral therapy. Exp Rev Cardiovasc Ther.

[5] Summers KM, Martin KE, Watson K (2010). Impact and clinical management of depression in patients with coronary artery disease. Pharmacotherapy.

[6] Mazereeuw G, Herrmann N, Bennett SA, Swardfager W, Xu H, Valenzuela N (2013). Platelet activating factors in depression and coronary artery disease: a potential biomarker related to inflammatory mechanisms and neurodegeneration. Neurosci Biobehav Rev.

[7] Celano CM, Millstein RA, Bedoya CA, Healy BC, Roest AM, Huffman JC (2015). Association between anxiety and mortality in patients with coronary artery disease: a meta-analysis. Am Heart J.

[8] Saeidi M, Soroush A, Komasi S, Brugnera A, Patucelli M, Carrozzino D (2018). Efficacy of alternative cardiac rehabilitation delivery formats in improving psychological symptoms after coronary artery bypass grafting. J Tehran Heart Cent.

[9] Hojskov IE, Moons P, Egerod I, Olsen PS, Thygesen LC, Hansen NV (2019). Early physical and psycho-educational rehabilitation in patients with coronary artery bypass grafting: A randomized controlled trial. J Rehab Med.

[10] Yang L, Wang X, Cui X (2019). Patients Intensive telephone-based care program reduces depression in coronary artery disease patients and may contribute to favorable overall survival by decreasing depression. J Cardiovasc Nurs.

[11] Ma L, Deng L, Yu H (2020). The effects of a comprehensive rehabilitation and intensive education program on anxiety, depression, quality of life, and major adverse cardiac and cerebrovascular events in unprotected left main coronary artery disease patients who underwent coronary artery bypass grafting. Ir J Med Sci.

[12] Huang K, Liu W, He D, Huang B, Xiao D, Peng Y (2015). Telehealth interventions versus center-based cardiac rehabilitation of coronary artery disease: a systematic review and meta-analysis. Eur J Prev Cardiol.

[13] Luo J, Dong X, Hu J (2019). Effect of nursing intervention via a chatting tool on the rehabilitation of patients after Total hip Arthroplasty. J Orthop Surg Res.

[14] Li X, Li T, Chen J, Xie Y, An X, Lv Y (2019). A WeChat-based self-management intervention for community middle-aged and elderly adults with hypertension in Guangzhou, China: a cluster-randomized controlled trial. Int J Environ Res Public Health.

[15] Grafting (2020). NCfCDECWCoCRACAB. Expert Consensus on Cardiac Rehabilitation After Coronary Artery Bypass Grafting [in Chinese]. Chinese Circulation Journal.

[16] Snaith RP, Zigmond AS (1986). The hospital anxiety and depression scale. Br Med J.

[17] Lam CL, Tse EY, Gandek B (2005). Is the standard SF-12 health survey valid and equivalent for a Chinese population?. Qua Life Res.

[18] Hillis LD, Smith PK, Anderson JL, Bittl JA, Bridges CR, Byrne JG (2011). 2011 ACCF/AHA guideline for coronary artery bypass graft surgery: a report of the American College of Cardiology Foundation/American Heart Association Task Force on Practice Guidelines. Circulation.

[19] Kang SH, Ahn JM, Lee CH, Lee PH, Kang SJ, Lee SW (2017). Differential event rates and independent predictors of long-term major cardiovascular events and death in 5795 patients with unprotected left main coronary artery disease treated with stents, bypass surgery, or medication: insights from a large International Multicenter Registry. Circ Cardiovasc interv.

[20] Dorje T, Zhao G, Scheer A, Tsokey L, Wang J, Chen Y (2018). SMARTphone and social media-based Cardiac Rehabilitation and Secondary Prevention (SMART-CR/SP) for patients with coronary heart disease in China: a randomised controlled trial protocol. BMJ Open.

[21] Huo T, Guo Y, Shenkman E, Muller K (2018). Assessing the reliability of the short form 12 (SF-12) health survey in adults with mental health conditions: a report from the wellness incentive and navigation (WIN) study. Health Qual Life Outcomes.

